# Supplementary material for: Interplay between a polerovirus and a closterovirus decreases aphid transmission of the polerovirus
Source: Microbiol Spectr. 2024 Oct 10;12(11):e01115-24. doi: 10.1128/spectrum.01115-24 (PMC11537018; doi:10.1128/spectrum.01115-24)
Supplement: Supplemental material — Fig. S1 and S2; Tables S1 and S2. [file spectrum.01115-24-s0005.pdf]

SUPPLEMENTS (Figures S1-S2, Tables S1-S2)

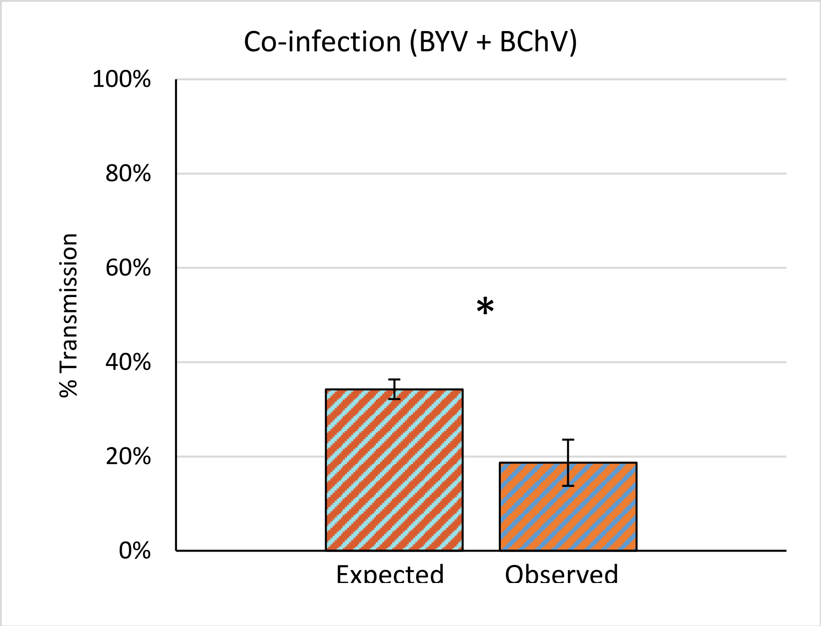

Figure S1. Aphid transmission efficiency of BYV and BChV from co-infected plants. *Myzus persicae* acquired virus from co-infected leaves for 24 h. Then, three aphids were transferred per test plant for 72 h inoculation. A DAS-ELISA assay was performed 3 weeks later to detect infection. The percentage of expected and observed co-infected plants is indicated. Difference was statistically significant (p-value= 0.02865; n=75, five independent experiments; df=1; chi-squared(χ2)).

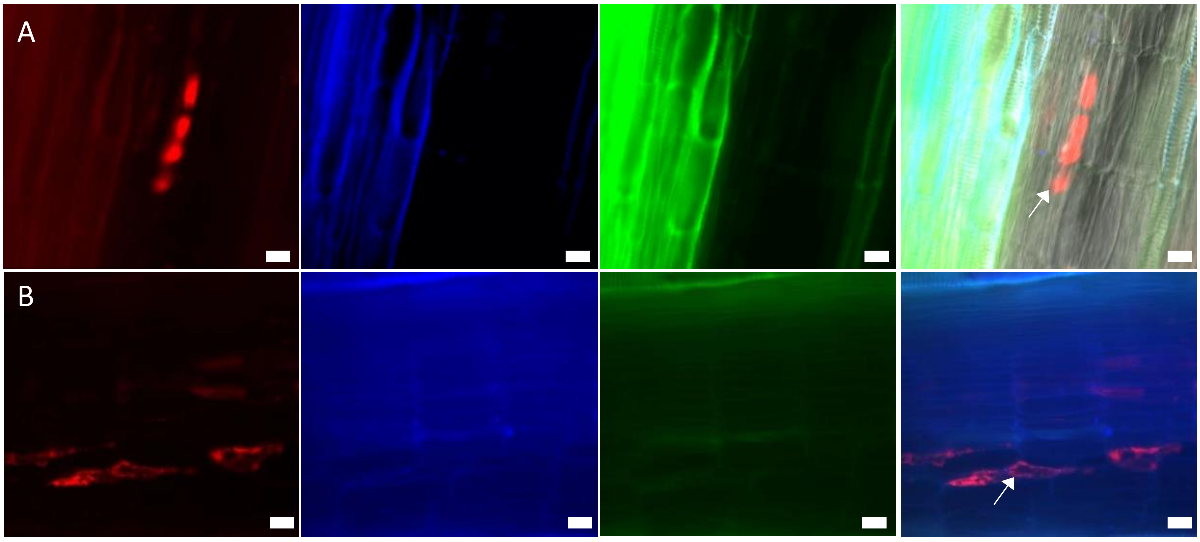

Figure S2. Tissue distribution of BChV and BYV in roots mono-infected with BChV or BYV. Sugar beet roots were processed three weeks after inoculation for immunofluorescence detection of BChV and BYV. (A-B) show longitudinal sections of (A) BYV mono-infected, (B) BChV mono-infected roots. The first column shows the virus label, the second and the third columns show autofluorescence and the last column presents merges. The white arrows in the image merges indicate the virus label. Scale bars = 200 µm (A-B).

Table S1. Primers with concatemer sequences (red) used for Primer Exchange Reaction.

BYV	
Primer1	CTGTCGGGCACAATGGGGCTTGCACTTTATAAACCTA
Primer2	CATGTCTCGGCGGGTGTCCACGACTTTATAAACCTA
Primer4	GGGTTGTCGGTAGAAGCACTCAAGTGCACACGTTTATAAACCTA
Primer5	GCACGACCTCGGTGCAGGCTCGTTTATAAACCTA
Primer6	GGGAACTGACAGAGAGGACTTCAGTTGGGAACACGTTTATAAACCTA
Primer7	CTGTAAGAGCCGACCAGCTGGAAGAGTCTTCGTTTATAAACCTA
Primer8	GGGCGCGAGTTAGGTTTCGATGTAACACCTAACGTTTATAAACCTA
Primer9	CGCGAGGAGTTTGTTGCCTCCTGCGTTTATAAACCTA
Primer10	CGCGCGCCTGAGCTTCAACAAGTTGTTCGTTTATAAACCTA
Primer11	GTTCGCCCGGAGTCACCATGGTGAGGTTTATAAACCTA
Primer12	GCCATGTCGATAGCGAGAGTTAAACGTTAAAGGCAGGGTTTATAAACCTA
Primer13	CACTCGAGTGTAAGAGACCGAACTGGGTTTCAACCTATTCATACTTTATAAACCTA
Primer14	CCATTTCGGCGTGCGTGGGAATTTGTGCATTTATAAACCTA
Primer15	CTACGAAAGGAGCCGCCACTTCAGCCAGTTCTTTATAAACCTATTTATAAACCTA
Primer16	CTTCCACTCCACTCATGTTTATTGTGAATTCACCGAGAGCCGTTTATAAACCTA
Primer17	GGAACAGTTTGGGCAACGAACGAGCTATCACTATGTAAGCTTTATAAACCTA
Primer18	CGGCGCGAACACTGGGTCCGAGTTTATAAACCTA
Primer19	GCGGCGTGGCCGCAGTGACTACTTTATAAACCTA
Primer 20	GAGCGGCGTGGGCGAGGTAACATAACCTTTATAAACCTA
Primer21	CGAGAGACTCGTCTTCCGCGCACAGGTTTATAAACCTA
Primer 22	GCTGACACGTTCGTAGACTGATTCGATCGCCTTGTTTATAAACCTA
Primer23	CCACTCTGCACGGCCTCCCCCATATAGAAGTTTATAAACCTA
Primer 24	CCAGCAGACGGGAACCGGCCGTTTATAAACCTA
Primer25	CGGTCTGCACACGTGGTAATCTGTGCTCCTTTATAAACCTA
Hairpin	Hairpin.26 : AATAAACCTAGGGCCTTTTGGCCCTAGGTTTATTTAGGTTTATTTTTTT
Imager	Fluor26 /ATTO647N/TTTAGGTTTATT TAGGTTTATT

BChV	
Primer 1	GTGCCATGGTCCCAAGAGCTCCAAATAAGATTAGGGTTTCATCATCAT
Primer 2	TGTAGCCTCTTTGCAAGACGTGTATTACAAGTTCTTTGTAGCTGTTTCATCATCAT
Primer 3	AGGCCATCAATGAGGCCACTATTATAGCCAGGCTTTCATCATCAT
Primer 4	GGGTTTCATTGGTACAGCCCATGTAGTGAACCCCTTTCATCATCAT
Primer 5	CGCGCTCATCACCATCCTTGCCGTTTCATCATCAT
Primer 6	GGTCTCTCTATGCAAACTCTCTTCATCGTGGTGAACGCTTTCATCATCAT
Primer 7	TCGTTTTGAAGCAAATCTCAAAGTTCATTCACTGTCCTCCTGCTTTTCATCATCAT

Primer 8	CAAGTGTCTTTCGCTCGTCACTAGTAGTTCGCTGTTTTCATCATCAT
Primer 9	GGTATCAACTTGGTGGACCAGTTATTGAAAACTTCTTGTGGTTCCTCACTTTCATCATCAT
Primer 10	GGACTTCCATGTCATCTTCGAGCATCCAGTCCGCTACTTTCATCATCAT
Primer 11	CGTAATCGTTTTGTTGTGTGGTTGTTGTTTCTGGTCAAGCGATTGCTTTCATCATCAT
Primer 12	GCTTGATCCTCCTGAATTAGTTTTGTGGTTGGACTGGAATGACCTTTCATCATCAT
Primer 13	GGTCTCCACGATTCAACCAATTCCGGCGTTGGATTAAAATTTCCTTTCATCATCAT
Primer 14	GGAGTTTTAAGGTCTTGTCTTTGACCGGTTTTGAGTTTCTCGGAGTCTTTCATCATCAT
Primer 15	CCAGTGACGGTTGAGAAAACACTCCCATTGATGGGTTTCATCATCAT
Primer 16	TGCGTGTGAACCGTCGTCATGACTTATGAGCTTTCATCATCAT
Hairpin	Hairpin.27 : ACATCATCATGGGCCTTTTGGCCCATGATGATGTATGATGATGTTTTTTT
Imager	Fluor 27 /5ATTO550N/TTATGATGATGTATGATGATGT

Table S2. Amplification efficacy (%) of reference genes, and the associated correlation coefficient (R^2).

Efficacy expected values according to literature (De Keyser et al., 2013; Broeders et al., 2014; Veselenak et al., 2015; Shehata et al., 2019; Stephan, Tilmes, et Hülskamp, 2019) and according to R^2 value.

Abbreviations: *PP2A*: Protein phosphatase 2A; *NbUBC*: Ubiquitin-conjugating enzyme 3; *NbTUB*: β-Tubulin; *NbLip*: Lipopyl synthase 2, mitochondria; *GAPDH*: Glyceraldehyde 3-phosphate dehydrogenase.

Gene	Efficacy (%)	R^2
**Expected values**	**80-120**	**≥ 0.980**
PP2A	101.5	0.996
NbUBC	101.8	0.995
NbTUB	106.5	0.997
NbLip	91	0.997
GAPDH	97.6	0.991
